# Effect of a Structured Physiotherapy Rehabilitation Program on Freezing of Gait, Mobility, and Balance in Patients With Stage III Chronic Parkinson’s Disease: A Case Report

**DOI:** 10.7759/cureus.113806

**Published:** 2026-08-02

**Authors:** Mukta T Killedar, Suraj B Kanase

**Affiliations:** 1 Department of Neurosciences Physiotherapy, Krishna Vishwa Vidyapeeth (Deemed to be University), Karad, IND

**Keywords:** balance, case report, freezing of gait, functional mobility, gait training, neurorehabilitation, parkinson’s disease, physiotherapy rehabilitation

## Abstract

Parkinson’s disease is a progressive neurodegenerative disorder that commonly results in gait dysfunction, balance impairment, postural instability, and reduced functional independence. Freezing gait (FOG) is among the most disabling motor manifestations of the disease and is associated with an increased risk of falls, impaired mobility, and reduced quality of life. Although exercise-based rehabilitation has demonstrated beneficial effects in individuals with Parkinson’s disease, detailed reports describing the response of patients with long-standing disease and significant freezing gait remain limited.

This case report describes the effects of a structured physiotherapy rehabilitation program in a 74-year-old male with a 15-year history of Stage III Parkinson’s disease who presented with freezing gait, shuffling gait, impaired balance, rigidity, tremor, postural abnormalities, and reduced functional mobility. The patient underwent a six-week rehabilitation program consisting of gait training, balance retraining, cueing strategies, strengthening exercises, flexibility exercises, postural correction exercises, aerobic conditioning, coordination training, and functional task practice for 45 minutes per session, five days per week. Outcomes were assessed using the Timed Up and Go (TUG) test, Berg Balance Scale (BBS), and Modified Hoehn and Yahr staging system.

Following the intervention, functional mobility improved, with TUG time decreasing from 21 seconds to 14 seconds. Balance performance improved, with the BBS score increasing from 28/56 to 41/56. The Modified Hoehn and Yahr stage changed from Stage III to Stage II following rehabilitation. Clinical observations also suggested reductions in freezing episodes, shuffling gait, rigidity, tremor, micrographia, hypophonia, and postural abnormalities. The patient additionally reported improved walking ability, greater confidence during ambulation, reduced fear of falling, and enhanced endurance during daily activities.

This case report highlights the potential benefits of a structured, multi-component physiotherapy rehabilitation program for improving freezing gait, functional mobility, balance, and overall functional performance in an individual with long-standing Parkinson’s disease. These findings support the role of individualized physiotherapy as an important adjunct to pharmacological management in chronic Parkinson’s disease. However, these findings should be interpreted cautiously and should not be interpreted as evidence of reversal of the underlying neurodegenerative disease.

## Introduction

Parkinson's disease (PD) is a progressive neurodegenerative disorder characterized by degeneration of dopaminergic neurons within the substantia nigra, resulting in impaired basal ganglia function and progressive deterioration of motor control [1,2]. Clinically, Parkinson's disease presents with motor manifestations including bradykinesia, muscular rigidity, resting tremor, postural instability, and gait dysfunction, which contribute to impaired mobility, reduced functional independence, and diminished quality of life. In addition to these motor manifestations, individuals frequently experience non-motor symptoms such as cognitive impairment, mood disturbances, sleep disorders, and autonomic dysfunction, further increasing disease burden and disability [1]. The global prevalence of Parkinson's disease has increased substantially over recent decades, creating a significant healthcare and socioeconomic burden worldwide [3]. Disease severity is commonly classified using the Modified Hoehn and Yahr (mH&Y) staging system, with advancing stages associated with greater gait impairment, balance deficits, and progressive functional limitations [4,5]. The mH&Y staging system is widely used to classify the overall functional severity and progression of Parkinson's disease based on postural stability and ambulatory capacity. However, it was primarily developed as a disease staging tool rather than a measure of short-term rehabilitation outcomes. Consequently, changes observed over a brief rehabilitation period should be interpreted as reflecting improvements in functional clinical status rather than reversal of the underlying neurodegenerative process [4,5].

Among the motor manifestations of Parkinson's disease, freezing gait (FOG) is one of the most disabling and clinically challenging symptoms. It is characterized by brief episodes during which an individual experiences difficulty initiating or continuing walking despite the intention to move, particularly during gait initiation, turning, or negotiating narrow spaces [6]. Freezing gait is associated with an increased risk of falls, impaired community mobility, reduced independence, and poorer quality of life, with its frequency and severity increasing as the disease progresses [6-8]. Individuals with long-standing Stage III Parkinson's disease frequently present with postural instability, impaired balance, gait dysfunction, and persistent freezing gait, substantially increasing fall risk and limiting functional independence. Rehabilitation in this stage is particularly challenging because progressive motor impairment, reduced automatic movement control, impaired postural responses, and decreased adaptability to environmental demands often restrict mobility despite optimized pharmacological management. Although pharmacological therapy remains the cornerstone of Parkinson's disease management, medications alone often fail to adequately address gait dysfunction, postural instability, balance impairment, and freezing episodes. Consequently, physiotherapy has become an essential component of multidisciplinary rehabilitation, aiming to improve mobility, postural control, gait performance, balance, and overall functional independence [9].

A growing body of evidence supports the role of exercise-based rehabilitation in improving motor function and functional performance in individuals with Parkinson's disease. Structured physiotherapy interventions, including treadmill training, progressive resistance exercise, task-specific training, cueing strategies, balance training, aerobic exercise, Pilates, hydrotherapy, and other multimodal rehabilitation approaches, have demonstrated beneficial effects on gait performance, balance, muscle strength, mobility, and overall physical function [10-19]. Functional outcomes following rehabilitation are commonly evaluated using validated assessment tools such as the Timed Up and Go (TUG) test and the Berg Balance Scale (BBS), while overall functional disease stage is commonly classified using the mH&Y staging system [4,20-22]. Although previous studies have demonstrated the effectiveness of individual rehabilitation interventions and multimodal exercise programs in Parkinson's disease, most have focused on group-based clinical studies or specific exercise modalities rather than providing detailed descriptions of individualized rehabilitation in patients with long-standing stage III Parkinson's disease and prominent FOG. Consequently, published case reports describing the implementation and outcomes of a comprehensive, multi-component physiotherapy rehabilitation program in this population remain limited, highlighting the clinical relevance and novelty of the present report [9-19,23].

Therefore, this case report describes the effects of a six-week structured physiotherapy rehabilitation program comprising balance training, gait training, cueing strategies, strengthening exercises, flexibility exercises, postural correction exercises, functional task training, aerobic training, and coordination exercises in a patient with a 15-year history of stage III Parkinson's disease presenting with long-standing FOG. This case is clinically noteworthy because it demonstrates the implementation of an individualized, multi-component rehabilitation program in a patient with advanced functional impairment and illustrates meaningful improvements in functional mobility, balance, FOG, and overall functional clinical status following rehabilitation. Outcomes were evaluated using the Timed Up and Go (TUG) test, Berg Balance Scale (BBS), and mH&Y staging system to assess changes in freezing gait, functional mobility, balance, and functional clinical status following the six-week intervention [4,20,21].

## Case presentation

A 74-year-old male presented to the Neurosciences Outpatient Department of Krishna College of Physiotherapy (Karad, MH, IND) with complaints of progressive difficulty in walking, episodes of FOG, impaired balance, shuffling gait, reduced voice volume, and difficulty performing functional mobility activities independently. The patient had a 15-year history of Parkinson’s disease and had been diagnosed and managed by a neurologist. Despite ongoing medical treatment, he reported gradual deterioration in mobility, gait performance, and postural stability, which negatively affected his independence and community participation. The patient sought physiotherapy rehabilitation because of progressive gait dysfunction and balance impairment that interfered with activities of daily living and safe ambulation. He had not undergone any previous structured physiotherapy rehabilitation before presentation to our department.

The patient also had a history of hypertension and type 2 diabetes mellitus, both of which were medically managed. The patient was receiving routine pharmacological management for Parkinson’s disease consisting of levodopa/carbidopa (Syndopa Plus), one tablet three times daily before meals, and trihexyphenidyl 1 mg twice daily. No changes were made to the patient's antiparkinsonian medication regimen during the intervention period. Despite ongoing pharmacological treatment, he continued to experience FOG, impaired balance, gait dysfunction, and reduced functional mobility. There was no history of falls; however, the patient required a cane for community ambulation because of gait instability and impaired balance.

Clinical examination revealed multiple motor manifestations of Parkinson’s disease, including bradykinesia, generalized rigidity, pill-rolling resting tremor, shuffling gait, and episodes of freezing, particularly during gait initiation and turning. Neurological examination demonstrated reduced arm swing during walking, impaired postural reactions, decreased movement amplitude, and difficulty performing coordinated functional movements. Cognitive function was adequate to understand verbal instructions and actively participate in the rehabilitation program.

Postural assessment demonstrated a stooped posture with forward head alignment and a positive floating pillow sign suggestive of axial rigidity and impaired postural control. Additional clinical findings included micrographia and hypophonia. The patient demonstrated impaired balance, reduced gait efficiency, and difficulty performing functional mobility activities such as walking, turning, and transfers independently.

Disease severity was assessed using the mH&Y staging system [4] based on clinical evaluation of postural stability and functional mobility, and the patient was classified as stage III at baseline. Functional mobility was assessed using the TUG test [20], which demonstrated a completion time of 21 seconds, indicating impaired mobility and an increased risk of falls. Balance was assessed using the Berg Balance Scale (BBS) [21], yielding a score of 28 out of 56, reflecting significant balance impairment and postural instability. All baseline and follow-up outcome assessments, including mH&Y staging, were performed by the treating physiotherapist during the patient's medication-ON state before the rehabilitation session to minimize the influence of motor fluctuations and ensure consistency between assessments. All baseline outcome assessments were performed during the patient's medication-ON state before initiation of the rehabilitation program to minimize the influence of motor fluctuations and ensure consistency of assessment.

Intervention

The intervention was selected because the patient presented with FOG, impaired balance, postural instability, gait dysfunction, and reduced functional mobility despite ongoing pharmacological management. A structured physiotherapy rehabilitation program was prescribed to address these impairments and improve functional independence.

The patient underwent a six-week physiotherapy rehabilitation program consisting of gait training, balance training, external cueing strategies, strengthening exercises, flexibility exercises, postural correction exercises, aerobic conditioning, coordination training, and functional task practice. Treatment sessions were conducted for 45 minutes per day, five days per week under the supervision of a qualified physiotherapist. The detailed components of the structured physiotherapy rehabilitation program are presented in Table 1.

**Table 1:** Components, dosage, and progression of the structured physiotherapy rehabilitation program

Intervention component	Description	Dosage/progression
Balance training	Static and dynamic balance activities	Eight to 10 minutes; progressed according to patient stability and safety response
Gait training with dual-task activities	Walking combined with cognitive or motor tasks and cueing strategies	Eight to 10 minutes; progressed according to patient tolerance and safety
Resistance training	Moderate-intensity strengthening exercises for lower limbs and trunk	Two sets of 10 repetitions
Core stabilization exercises	Controlled breathing, trunk control, and postural stabilization exercises	Two sets of 10 repetitions
Functional strengthening	Sit-to-stand practice, squatting, and lifting activities	Two sets of 10 repetitions
Coordination training	Rhythmic and patterned movement activities	Five to seven minutes; progressed according to performance capacity and movement quality
Flexibility exercises	Stretching exercises for muscles affected by rigidity and reduced mobility	Two repetitions with 20 to 30-second hold for each major muscle group
Frequency and duration	Supervised physiotherapy rehabilitation program	Total session duration: 45 minutes; five days per week for six weeks

The rehabilitation program was individualized according to the patient’s clinical presentation and functional limitations. Gait training emphasized stride length, turning, movement initiation, and walking efficiency. External auditory and visual cueing strategies were incorporated to facilitate movement initiation and reduce freezing episodes. Balance training progressed from static to dynamic activities according to patient performance and safety. Strengthening exercises focused on lower-limb, trunk, and postural musculature, while flexibility exercises targeted muscle groups affected by rigidity and reduced mobility. Coordination exercises and functional task training were incorporated to improve movement control and performance of daily activities.

At the beginning of rehabilitation, the patient demonstrated impaired balance, reduced gait efficiency, and frequent freezing episodes during gait initiation and turning. Therefore, exercises were initially performed at a level that ensured patient safety while emphasizing movement quality and postural control. As mobility and confidence improved, exercise difficulty was progressively increased through greater balance challenges, increased gait complexity, dual-task activities, and advancement of functional mobility tasks. Progression of the rehabilitation program was guided by patient tolerance, movement quality, balance performance, fatigue level, and overall clinical response. The patient completed the intervention program without adverse events and demonstrated good adherence to the prescribed treatment sessions.

Outcomes

Following completion of the six-week rehabilitation program, reassessment demonstrated clinically meaningful improvements in mobility, balance, and disease severity. Clinical outcomes before and after the intervention are summarized in Table 2. Functional mobility improved, with the TUG test time decreasing from 21 seconds at baseline to 14 seconds following the intervention. Balance performance also improved substantially, with the BBS score increasing from 28/56 to 41/56. The patient's mH&Y stage changed from stage III at baseline to stage II following rehabilitation. All baseline and follow-up outcome assessments, including mH&Y staging, were performed during the patient's medication-ON state by the treating physiotherapist.

**Table 2:** Comparison of clinical outcome measures before and after the six-week structured physiotherapy rehabilitation program TUG: Timed-Up and Go, BBS: Berg Balance Scale, mH&Y: Modified Hoehn and Yahr

Outcome measure	Pre-intervention	Post-intervention
TUG test (seconds)	21	14
BBS score	28	41
mH&Y staging	III	II

Clinically, the patient demonstrated improved gait performance, reduced frequency of freezing episodes, enhanced balance, improved postural control, and increased confidence during ambulation with a cane. Improvements were also observed in movement initiation, turning ability, and overall functional mobility. Furthermore, the patient reported greater ease in performing transfers, walking-related activities, and community ambulation compared with baseline.

Overall, the six-week structured physiotherapy rehabilitation program was associated with clinically meaningful improvements in FOG, functional mobility, balance, and postural control. The observed change in the mH&Y stage represented a change in functional clinical status following rehabilitation and should not be interpreted as reversal of the underlying neurodegenerative disease.

## Discussion

This case report describes the clinical outcomes of a six-week structured physiotherapy rehabilitation program in a 74-year-old male with a 15-year history of Parkinson's disease. At baseline, the patient presented with Stage III disease, freezing gait, impaired balance, shuffling gait, rigidity, resting tremor, postural abnormalities, and reduced functional mobility. Following the intervention, clinically meaningful improvements were observed across multiple domains. Functional mobility improved, as demonstrated by a reduction in TUG time from 21 seconds to 14 seconds, while balance performance improved with an increase in BBS score from 28/56 to 41/56. The patient's mH&Y stage changed from stage III at baseline to stage II following rehabilitation. Clinical observations also demonstrated reductions in freezing episodes, shuffling gait, rigidity, tremor, micrographia, hypophonia, and postural abnormalities. Additionally, the patient reported improved walking ability, increased endurance, greater confidence during ambulation, and enhanced independence in performing activities of daily living.

The favorable outcomes observed in this patient are likely attributable to the comprehensive and individualized nature of the rehabilitation program. Although Parkinson's disease is a progressive neurodegenerative disorder, exercise-based rehabilitation has been shown to promote adaptive neuroplastic changes that may improve motor learning, movement control, and functional performance [10]. Consequently, physiotherapy has become an important adjunct to pharmacological management, particularly for addressing gait dysfunction, postural instability, balance impairment, and freezing gait that often persist despite optimal medical therapy [9,23].

One of the most important findings of this case was the reduction in FOG. Freezing of gait is among the most disabling manifestations of Parkinson's disease and is associated with impaired mobility, increased fall risk, reduced independence, and poorer quality of life [6-8]. In this patient, external cueing strategies, including verbal commands, floor markings, and counting techniques, were incorporated throughout gait training. These interventions are thought to facilitate movement initiation by compensating for impaired internal cueing mechanisms and recruiting alternative neural pathways involved in motor control [24]. Previous studies have similarly reported that cue-based rehabilitation improves gait initiation, movement amplitude, and overall mobility in individuals with Parkinson's disease [15,16,24]. The reduction in freezing episodes observed in this patient is consistent with these findings.

Meaningful improvements were also observed in functional mobility and balance following rehabilitation. The reduction in TUG time and increase in BBS score indicate enhanced walking ability, postural control, and functional independence. These improvements are likely attributable to the combined effects of gait training, balance retraining, strengthening exercises, flexibility exercises, postural correction, aerobic conditioning, coordination training, and functional task practice incorporated into the rehabilitation program. Previous studies have demonstrated that treadmill training, progressive resistance exercise, task-specific training, Pilates, hydrotherapy, and other structured physiotherapy interventions improve gait performance, balance, muscle strength, and overall physical function in individuals with Parkinson's disease [11-19]. The findings of the present case are consistent with these reports and further support the role of multimodal physiotherapy in improving mobility and balance in individuals with chronic Parkinson's disease.

An important feature of the rehabilitation program was the gradual progression of exercises according to the patient's functional abilities and tolerance. Initially, interventions focused on safe performance of balance and mobility activities. As the patient's performance improved, exercise difficulty was progressively increased through greater balance challenges, dual-task activities, more complex gait tasks, and advanced functional exercises. Progressive task-specific training is recognized as an important principle of neurological rehabilitation because it promotes motor learning, enhances sensorimotor adaptation, and improves movement efficiency through repetitive practice [10,24]. The individualized progression of treatment likely contributed to the functional improvements observed during the rehabilitation period.

Clinical observations also demonstrated improvements in postural alignment, movement initiation, gait quality, and overall functional performance. Improvements in lower-limb strength, trunk stability, and postural control may have contributed to enhanced transfer ability, improved walking efficiency, and greater confidence during ambulation. The patient also reported increased confidence while walking, which may reflect a reduction in fear of falling. Fear of falling is common among individuals with Parkinson's disease and has been associated with activity limitation, reduced participation, and diminished quality of life [25]. Therefore, improvements in mobility and balance may have contributed to both physical and psychological benefits.

Another noteworthy finding was the change in the mH&Y stage from stage III to stage II following the rehabilitation program. The mH&Y staging system is primarily intended to classify functional disease severity rather than detect short-term rehabilitation outcomes [4]. Therefore, the observed change should be interpreted cautiously as reflecting improvements in functional mobility, postural stability, and overall functional status observed during the medication-ON state rather than reversal of the underlying neurodegenerative pathology or disease progression associated with Parkinson's disease.

The present case demonstrates that meaningful functional gains can be achieved even in an individual with long-standing Parkinson's disease. Despite a disease duration of 15 years and significant impairments in gait, balance, and functional mobility, substantial improvements were observed following six weeks of individualized rehabilitation. These findings support the growing body of evidence that structured physiotherapy interventions can complement pharmacological management by improving mobility, balance, functional performance, and quality of life in individuals with Parkinson's disease [9-19,23,26]. The present report also highlights the potential value of comprehensive rehabilitation programs that combine gait training, balance retraining, strengthening exercises, flexibility exercises, postural correction, aerobic conditioning, coordination training, and cueing strategies to address multiple motor impairments simultaneously.

Several limitations should be considered when interpreting the findings of this case report. As this report describes a single patient, the results cannot be generalized to the broader Parkinson's disease population. Multiple intervention components were delivered concurrently, making it difficult to determine the individual contribution of each component to the observed improvements. A disease-specific assessment tool for freezing gait, such as the FOG questionnaire (FOG-Q), was not administered because it was not included in the original clinical assessment protocol. Consequently, changes in FOG were evaluated based on detailed clinical observation during functional mobility tasks rather than a standardized disease-specific outcome measure. Similarly, objective motor symptoms, including bradykinesia, rigidity, and tremor, were evaluated through routine clinical examination and observation because the Movement Disorder Society-Unified Parkinson's Disease Rating Scale (MDS-UPDRS) part III was not included in the original assessment protocol. Long-term follow-up was also not performed to determine the sustainability of the observed improvements. Future studies involving larger sample sizes, standardized freezing gait assessments, quantitative motor outcome measures such as the MDS-UPDRS Part III, and extended follow-up are warranted to further evaluate the long-term effectiveness of comprehensive physiotherapy rehabilitation programs for individuals with chronic Parkinson's disease.

## Conclusions

This case report demonstrates that a structured physiotherapy rehabilitation program incorporating gait training, balance retraining, cueing strategies, strengthening exercises, flexibility exercises, postural correction, aerobic conditioning, and functional task practice was associated with meaningful improvements in FOG, functional mobility, balance, postural control, and overall functional performance in a patient with long-standing stage III Parkinson’s disease. Clinical observations also suggested improvements in gait performance, movement initiation, and patient-reported confidence during daily activities. These findings support the potential value of individualized, multi-component physiotherapy rehabilitation as an adjunct to pharmacological management for improving functional outcomes in individuals with chronic Parkinson’s disease. However, the findings should be interpreted cautiously because this report describes a single patient and should not be interpreted as evidence of reversal of the underlying neurodegenerative pathology of Parkinson’s disease. Further studies involving larger sample sizes, standardized outcome measures, and long-term follow-up are warranted to confirm the effectiveness and long-term sustainability of comprehensive physiotherapy rehabilitation programs.
